# Novel electrochemical platform based on C_3_N_4_-graphene composite for the detection of neuron-specific enolase as a biomarker for lung cancer

**DOI:** 10.1038/s41598-024-56784-x

**Published:** 2024-03-16

**Authors:** Zhang Junping, Wei Zheng, Tang ZhengFang, L. I. Ji Yue, An PengHang, Zhang Mingli, An Hongzhi

**Affiliations:** 1 Cancer Research Institute, Henan Integrative Medicine Hospital 45000, Zhengzhou, China; 2 The First School of Clinical Medicine, Henan University of Chinese Medicine, Zhengzhou, 450004 China

**Keywords:** Electrochemistry, Biomarkers, Diagnostic markers, Oncology, Cancer, Lung cancer

## Abstract

Lung cancer remains the leading cause of cancer mortality worldwide. Small cell lung cancer (SCLC) accounts for 10–15% of cases and has an overall 5-years survival rate of only 15%. Neuron-specific enolase (NSE) has been identified as a useful biomarker for early SCLC diagnosis and therapeutic monitoring. This work reports an electrochemical immunosensing platform based on a graphene-graphitic carbon nitride (g-C_3_N_4_) nanocomposite for ultrasensitive NSE detection. The g-C_3_N_4_ nanosheets and graphene nanosheets were synthesized via liquid exfoliation and integrated through self-assembly to form the nanocomposite. This nanocomposite was used to modify screen-printed carbon electrodes followed by covalent immobilization of anti-NSE antibodies. The unique properties of the graphene-g-C_3_N_4_ composite facilitated efficient antibody loading while also enhancing electron transfer efficiency and electrochemical response. Systematic optimization of experimental parameters was performed. The immunosensor exhibited a wide linear detection range of 10 pg/mL to 100 ng/mL and low limit of detection of 3 pg/mL for NSE along with excellent selectivity against interferences. Real serum matrix analysis validated the applicability of the developed platform for sensitive and accurate NSE quantifica-tion at clinically relevant levels. This novel graphene-g-C_3_N_4_ nanocomposite based electro-chemical immunoassay demonstrates great promise for early diagnosis of SCLC.

## Introduction

Lung cancer remains one of the most commonly diagnosed cancers and the leading cause of cancer-related deaths worldwide. In 2018 alone, over 2 million new lung cancer cases and 1.76 million lung cancer deaths were reported globally^1^. Non-small cell lung cancer (NSCLC) and small cell lung cancer (SCLC) are the two major histological subtypes, with NSCLC accounting for approximately 85% and SCLC accounting for 10–15% of lung cancer cases^2^. Despite advancements in treatment modalities over the past few decades, the overall 5-years survival rate for lung cancer patients remains poor at around 15%^3^. The high mortality rate associated with lung cancer is mainly attributed to the fact that majority of cases are diagnosed at an advanced stage when curative surgical treatment is no longer an option^4^. Therefore, there is an urgent need for developing effective strategies for early diagnosis to improve clinical outcomes.

The analysis of biomarkers present in body fluids such as blood offers a promising non-invasive approach for early lung cancer diagnosis. Neuron-specific enolase (NSE), a dimeric glycolytic isoenzyme found mainly in neuronal and neuroendocrine tissues, has been identified as a useful biomarker for SCLC diagnosis and monitoring^5^. Serum NSE levels are often elevated in SCLC patients at the time of diagnosis and can provide prognostic information during treatment^6^. Compared to other conventional tumor markers like carcinoembryonic antigen (CEA) and cytokeratin fragment 21–1 (CYFRA 21–1), NSE has higher sensitivity and specificity for SCLC^7^. A meta-analysis of 40 studies found that NSE had a sensitivity of 0.60 and specificity of 0.95 for differentiating SCLC from benign lung diseases^8^. Therefore, sensitive detection of serum NSE levels can facilitate early SCLC diagnosis and timely therapeutic intervention.

In recent years, electrochemical biosensors have emerged as promising tools for protein biomarker detection owing to their high sensitivity, simple instrumentation, low cost, and capability for rapid analysis^9–11^. Such biosensors utilize specific biorecognition elements like antibodies, aptamers or enzymes that can selectively capture biomarkers on transducer surfaces and translate molecular binding events into measurable electrical signals^12^. Graphene and graphene-based nanomaterials have attracted tremendous interest for electrochemical biosensing applications due to their high surface area, excellent conductivity, biocompatibility and ease of surface modification^13–17^. However, pristine graphene often suffers from aggregation and restacking issues which can adversely affect its sensing performance. Incorporating graphene with other nanomaterials to form three-dimensional hierarchical composites has been shown as an effective strategy to overcome this limitation^18^.

Graphitic carbon nitride (g-C_3_N_4_), a emerging metal-free semiconductor composed of carbon and nitrogen, has recently drawn much attention due to its intriguing electronic, optical and catalytic properties^19^. g-C_3_N_4_ possesses a layered structure similar to graphene along with abundant surface nitrogen defects that can facilitate molecular adsorption^20^. A few recent studies have demonstrated the potential of nanostructured g-C_3_N_4_ for electrochemical sensing of cancer biomarkers and other analytes^21–23^. However, the integration of g-C_3_N_4_ with graphene for electrochemical biosensing has not been widely explored.

In this work, we report the development of a g-C_3_N_4_-graphene nanocomposite based electrochemical immunosensing platform for ultrasensitive detection of NSE as a model protein biomarker. Ultrathin g-C_3_N_4_ nanosheets were synthesized by a simple liquid exfoliation approach and then integrated with graphene via self-assembly. The as-prepared nanocomposite was used to modify screen-printed carbon electrodes followed by covalent immobilization of anti-NSE antibodies. The unique properties of g-C_3_N_4_-graphene composite facilitated efficient antibody loading and sensitive electrochemical transduction of immunointeractions. Key parameters influencing the immunosensor performance were systematically optimized. Under optimal conditions, the proposed immunosensing platform exhibited wide dynamic range, low detection limit and high selectivity for quantitative detection of NSE, demonstrating its potential for early diagnosis of SCLC.

## Methods

### Chemicals and materials

Graphite powder was obtained from Alfa Aesar (USA). Melamine, dopamine hydrochloride, uric acid, potassium hexacyanoferrate(III), and all other reagents were purchased from Sigma-Aldrich (USA). Screen-printed carbon electrodes (SPCEs) were acquired from DropSens (Spain). Anti-neuron specific enolase (Anti-NSE) monoclonal antibodies and NSE antigen were purchased from Abcam (USA). Phosphate buffered saline (PBS, 0.1 M, pH 7.4) was prepared by mixing stock solutions of NaH_2_PO_4_ and Na_2_HPO_4_ and was used as the supporting electrolyte. All aqueous solutions were prepared with deionized (DI) water.

### Synthesis of g-C_3_N_4_ nanosheets

g-C_3_N_4_ nanosheets were synthesized by thermal polymerization of melamine followed by liquid exfoliation^24^. In brief, 5 g of melamine powder was put in an alumina crucible with a cover and heated at 550 °C for 2 h in air at a ramp rate of 2 °C/min using a muffle furnace (Thermo Scientific, USA). The resultant yellow agglomerates were ground into fine powder and dispersed in 100 mL water. The suspension was ultrasonicated using a probe sonicator (Qsonica Q125, USA) at 40% amplitude for 1 h with pulse on/off time of 5 s/2 s. The resultant mixture was centrifuged at 5000 rpm for 10 min to remove unexfoliated material. The supernatant containing exfoliated g-C_3_N_4_ nanosheets was carefully collected and dried overnight at 60 °C.

### Preparation of graphene nanosheets

Graphene nanosheets were synthesized by ultrasonic exfoliation of graphite^25^. 1 g of graphite powder was dispersed in a solution containing 400 mL water, 600 mL ethanol and 15 mg/mL dopamine hydrochloride. The mixture was ultrasonicated using a probe sonicator at 40% amplitude for 1 h. After centrifugation at 5000 rpm for 30 min, the top two-thirds of the supernatant was collected and vacuum filtered using a 0.22 μm pore polyvinylidene fluoride (PVDF) membrane to obtain graphene nanosheets.

### Fabrication of g-C_3_N_4_-graphene nanocomposite

The g-C_3_N_4_-graphene nanocomposite was prepared by mixing the as-prepared dispersions of g-C_3_N_4_ nanosheets and graphene nanosheets^26^. Typically, 20 mL of graphene dispersion (0.1 mg/mL) was added dropwise to 20 mL of g-C_3_N_4_ dispersion (0.1 mg/mL) under vigorous stirring. The mixture was further ultrasonicated for 30 min and then centrifuged and dried to obtain the final nanocomposite.

### Electrode modification

Prior to modification, SPCEs were cleaned by immersion in ethanol and DI water for 5 min each and dried with nitrogen. The g-C_3_N_4_-graphene nanocomposite dispersion was prepared in DI water (1 mg/mL) and 10 μL was drop cast onto the SPCEs followed by drying overnight at room temperature. For comparison, electrodes modified with only graphene were also fabricated.

### Immobilization of anti-NSE antibodies

**Figure 1:**
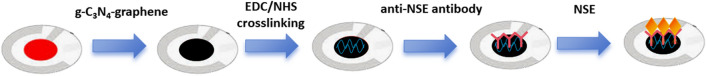
Fabrication process of proposed electrochemical sensor.

Covalent immobilization of antibodies was performed via EDC/NHS crosslinking^27^. 10 mM EDC and 10 mM NHS were freshly prepared in 0.1 M PBS (pH 7.4). 10 μL of the mixed EDC/NHS solution was drop cast onto the modified electrodes and incubated for 1 h to activate the carboxylic groups. The electrodes were then washed with PBS and 10 μL of anti-NSE antibody solution (10 μg/mL in PBS) was immobilized by incubating overnight at 4 °C. After rinsing off unbound antibodies, the electrodes were treated with 10 μL of 1% bovine serum albumin (BSA) for 1 h to block nonspecific binding sites. Finally, the resulting immunoelectrodes were washed with PBS and stored at 4 °C prior to use. Figure 1 shows the fabrication process of proposed electrochemical sensor. The ΔI value in the immunosensor response is calculated as the difference in the peak current obtained from differential pulse voltammetry before and after the addition of NSE antigen. Specifically, ΔI is determined as follows:

$$ \Delta {\text{I }} = {\text{ Ip}},{\text{before }} - {\text{ Ip}},{\text{after}} $$

where Ip,before is the peak current of the immunosensor measured in 0.1 M PBS (pH 7.4) prior to the addition of NSE antigen solution. This provides the baseline current.

Ip,after is the peak current obtained after incubating the immunosensor with a known concentration of NSE antigen solution for 1 h. The change in current occurs due to the immunoreaction between immobilized antibodies and target antigens on the electrode surface.

### Electrochemical measurements

All electrochemical measurements were performed on a CHI 660E workstation (CH Instruments, USA) using a conventional three-electrode system with the modified SPCE as working electrode, platinum wire counter electrode and Ag/AgCl (saturated KCl) reference electrode. Cyclic voltammetry (CV) scans were recorded in a redox probe solution containing 5 mM [Fe(CN)_6_]^3-/4−^ and 0.1 M KCl to characterize the electrode surface modification. Electrochemical impedance spectroscopy (EIS) was also performed in the same redox probe over a frequency range of 0.1 Hz to 100 kHz at open circuit potential with an AC perturbation of 5 mV.

For NSE detection, differential pulse voltammetry (DPV) technique was used in 0.1 M PBS (pH 7.4) at an amplitude of 50 mV, pulse width of 0.05 s and pulse period of 0.5 s. The immunoelectrodes were incubated with different concentrations of NSE antigen for 1 h at 37 °C followed by DPV analysis. The change in peak current response before and after immunoreaction was correlated to NSE concentration. Parameters such as incubation time, pH and temperature were optimized to obtain maximum sensor performance. Selectivity was evaluated by exposing the immunoelectrodes to potentially interfering proteins. At least triplicate measurements were performed for each concentration.

## Results and discussion

### Characterization of nanocomposite materials

**Figure 2:**
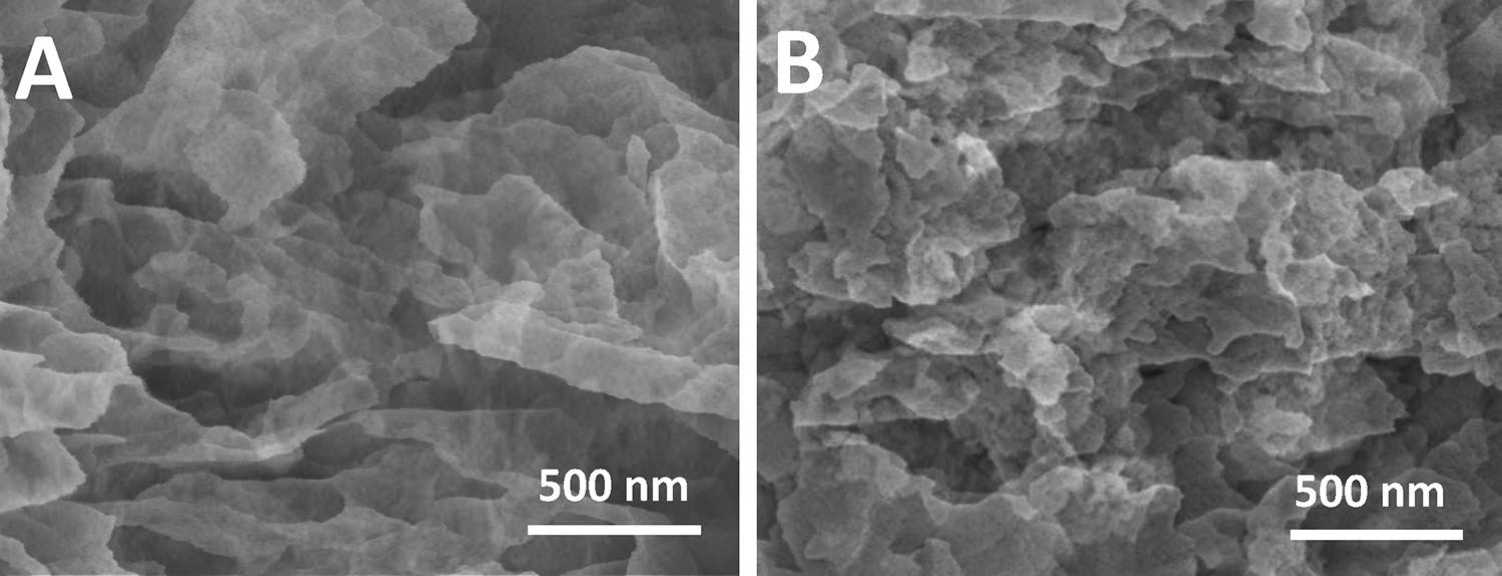
SEM image of (**A**) g-C_3_N_4_ nanosheets and (**B**) graphene-g-C_3_N_4_ composite.

The morphology of the synthesized g-C_3_N_4_ nanosheets and g-C_3_N_4_-graphene composite was examined by SEM using a FEI Nova NanoSEM 450 system. As shown in Fig. 2A, the SEM image of g-C_3_N_4_ nanosheets displays a thin, wrinkled sheet-like structure with lateral dimensions of 100–500 nm. The crumpled morphology indicates the successful exfoliation of g-C3N4 layers from the bulk material. The composite in Fig. 2B shows both graphene sheets and g-C_3_N_4_ nanosheets distributed uniformly without obvious aggregation.

**Figure 3:**
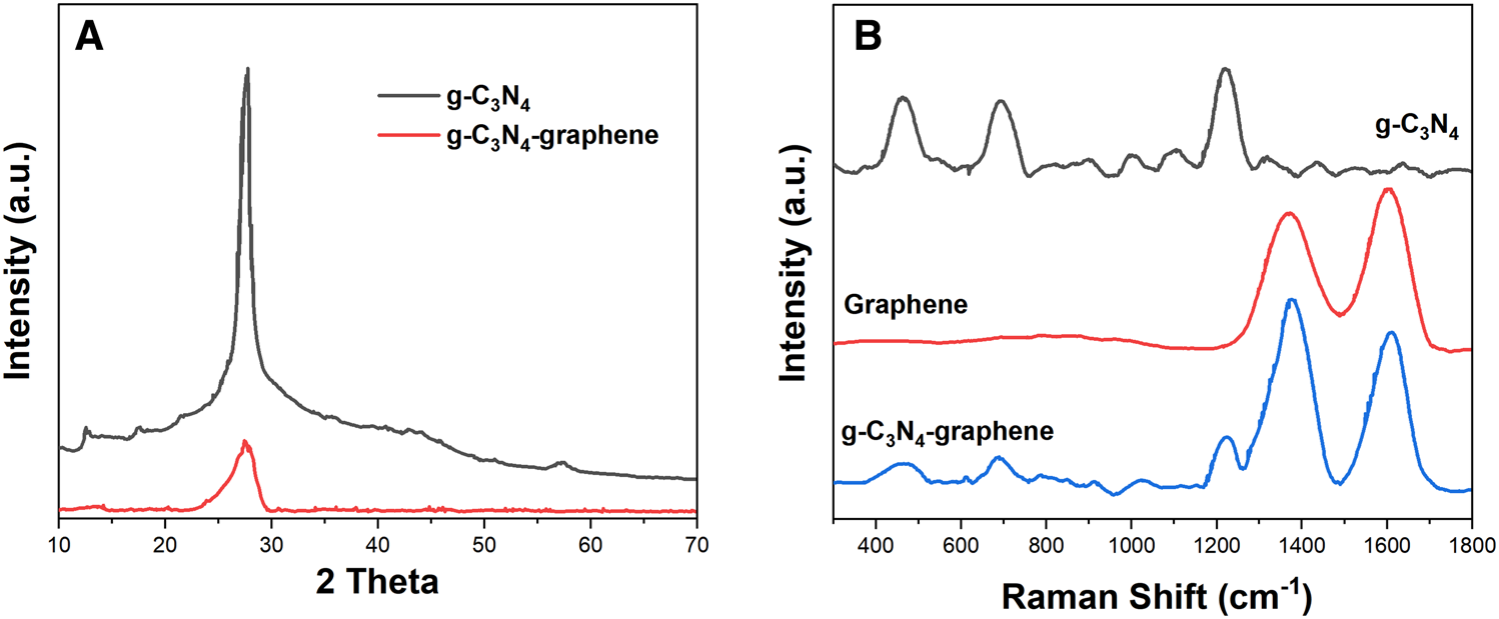
(**A**) XRD patterns of g-C_3_N_4_ nanosheets and graphene-g-C_3_N_4_ composite. (**B**) Raman spectra of g-C_3_N_4_ nanosheets, graphene and graphene-g-C_3_N_4_ composite.

XRD patterns were recorded on a Rigaku MiniFlex XRD system using Cu Kα radiation. For bulk g-C_3_N_4_, two characteristic diffraction peaks at 13.2° and 27.8° corresponding to the (100) and (002) lattice planes confirm the typical graphitic structure (Fig. 3A)^28^. In the pattern of exfoliated g-C_3_N_4_ nanosheets, the (002) peak is substantially diminished while the (100) peak is retained, suggesting the loss of interplanar stacking in the c-axis direction during exfoliation. The XRD pattern of the g-C_3_N_4_-graphene composite displays features of both components indicating the successful integration.

Raman spectroscopy was performed using a Horiba XploRA PLUS system with 532 nm laser excitation. As displayed in Fig. 3B, the spectrum of g-C_3_N_4_ nanosheets shows two prominent bands at 1240 cm^−1^ and 1566 cm^−1^ attributed to the C–N stretching vibration and typical G band of *sp*^2^ carbon domains respectively^29^. Graphene nanosheets exhibit the characteristic D band at 1352 cm^−1^ related to defects and G band at 1583 cm^−1^ corresponding to the E_2g_ phonon mode of *sp*^2^ carbon^30^. In the composite, all the signature peaks of g-C_3_N_4_ and graphene are observed confirming the presence of both components.

**Figure 4:**
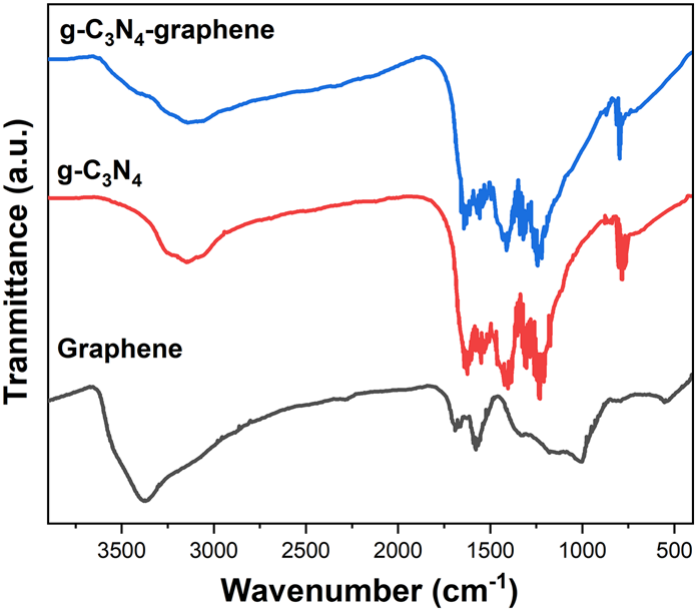
FTIR spectra of g-C_3_N_4_ nanosheets, graphene and graphene-g-C_3_N_4_ composite.

FTIR spectroscopy was done using a Thermo Scientific Nicolet iS5 spectrometer. The FTIR spectrum of g-C_3_N_4_ nanosheets (Fig. 4) displays characteristic peaks associated with the triazine units including 810 cm^−1^ (triazine ring vibration), 1240 cm^−1^ (C–N stretching), 1320 cm^−1^ (C–N heterocycle stretching) and 1635 cm^−1^ (C=N stretching)^31^. For graphene, the bands at 1060 cm^−1^, 1630 cm^−1^ and 3400 cm^−1^ correspond to C–O, C=C and O–H groups respectively^32^. In the composite spectrum, all the major vibrational modes of both components are present indicating successful hybridization. In contrast, the band at 1060 cm^−1^ of graphene has been hidden in the g-C_3_N_4_-graphene composite due to the surface of graphene and g-C_3_N_4_ combined to mask the signal of C–O. Similar phenomena have been observed in other papers that have been reported^33^.

### Characterization of biosensors

**Figure 5:**
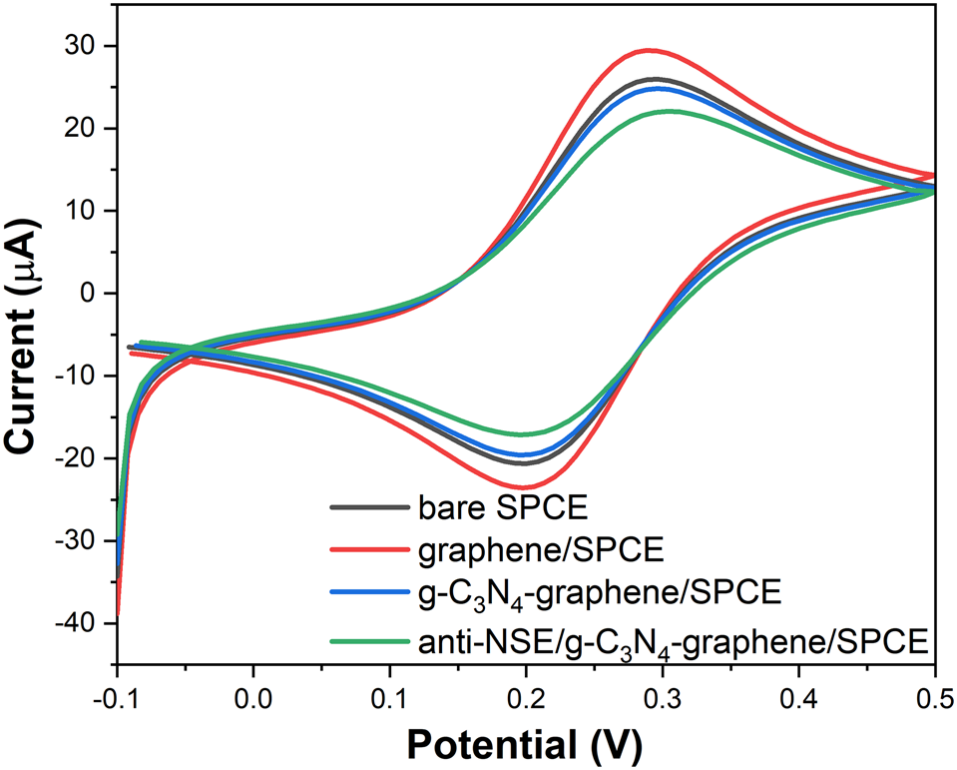
Cyclic voltammograms of bare SPCE, graphene/SPCE, g-C_3_N_4_-graphene/SPCE, and anti-NSE/g-C_3_N_4_-graphene/SPCE in 0.1 M PBS containing 5 mM [Fe(CN)_6_]^3−/4−^ at a scan rate of 50 mV/s.

CV was used to investigate the stepwise modification of the SPCEs with graphene, g-C_3_N_4_-graphene composite and anti-NSE antibodies. As shown in Fig. 5, a pair of well-defined redox peaks was observed for the bare SPCE due to the [Fe(CN)_6_]^3-/4-^ redox probe. After modification with graphene, the peak current increased significantly owing to the high charge transfer conductivity and large surface area. A decrease was seen with the g-C_3_N_4_-graphene composite due to the relatively non-conductive of g-C_3_N_4_^34^. After antibody immobilization, the peak current was further reduced indicating the inhibition of electron transfer by the insulating protein layer^35^. However, a distinct electrochemical response was still visible suggesting the composite provides a favorable microenvironment to retain biomolecule bioactivity^36^.

**Figure 6:**
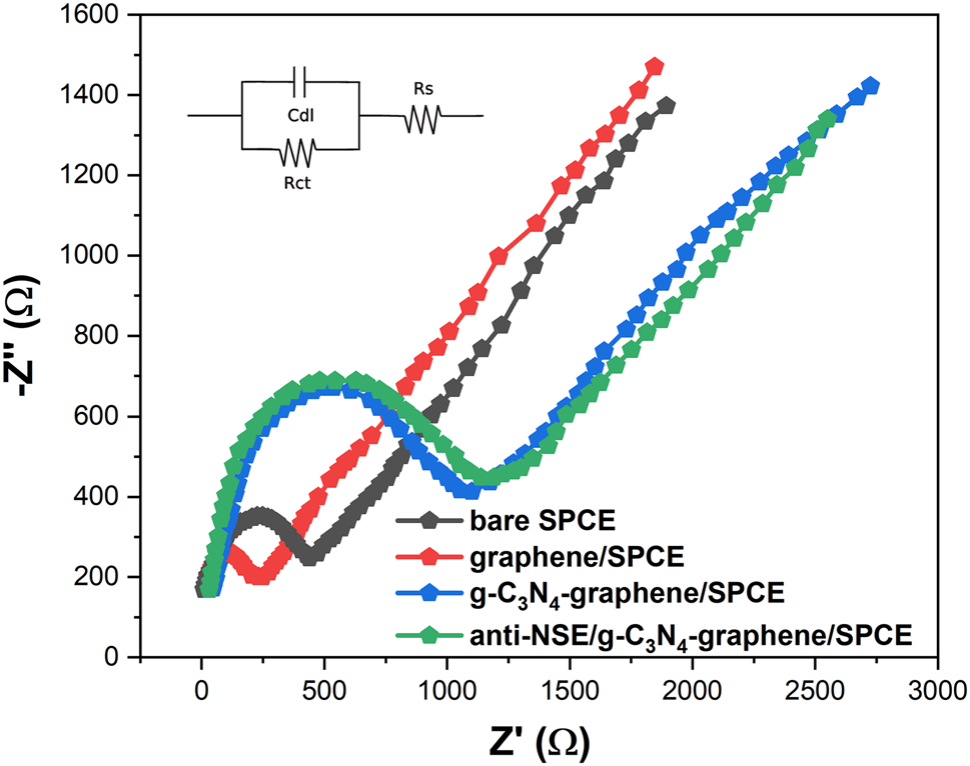
Nyquist plots obtained from electrochemical impedance spectroscopy of bare SPCE, graphene/SPCE, g-C_3_N_4_-graphene/SPCE, and anti-NSE/g-C_3_N_4_-graphene/SPCE in 0.1 M PBS containing 5 mM [Fe(CN)_6_]^3−/4−^. Inset shows the equivalent circuit model used for fitting the EIS data.

EIS was performed to characterize the interface properties. As displayed in Fig. 6, the unmodified SPCE showed a small semicircle domain representing low charge transfer resistance (Rct). The Rct value decreased after graphene modification due to its high conductivity. A significant increase of Rct was observed for the composite modified electrode^37^. The subsequent antibody immobilization led to a further increase in Rct, implying greatly inhibited electron transfer kinetics at the electrode interface. While the high surface area graphene-g-C_3_N_4_ composite allows for increased antibody loading, the antibodies are immobilized primarily on the outer surfaces and porous layers of the 3D nanostructure. This means there are still conductive pathways through the interior of the composite network that facilitate efficient electron transfer between the redox probe and the electrode interface. So the Rct change is less drastic compared to adding an insulating blocking layer across the entire electrode surface. The stepwise EIS changes confirm the successful modification and biofunctionalization processes. The integration of g-C_3_N_4_ with graphene forms a 3D porous network that prevents graphene sheet restacking, which allows for increased loading of capture antibodies on the sensor interface. So while some antibody binding sites may be less accessible due to diffusion limitations, the total number of antibodies immobilized is higher^33,38^. The results validate the development of the g-C_3_N_4_-graphene nanocomposite and its potential for electrochemical biosensing applications^39^.

### Optimization of biosensors

**Figure 7:**
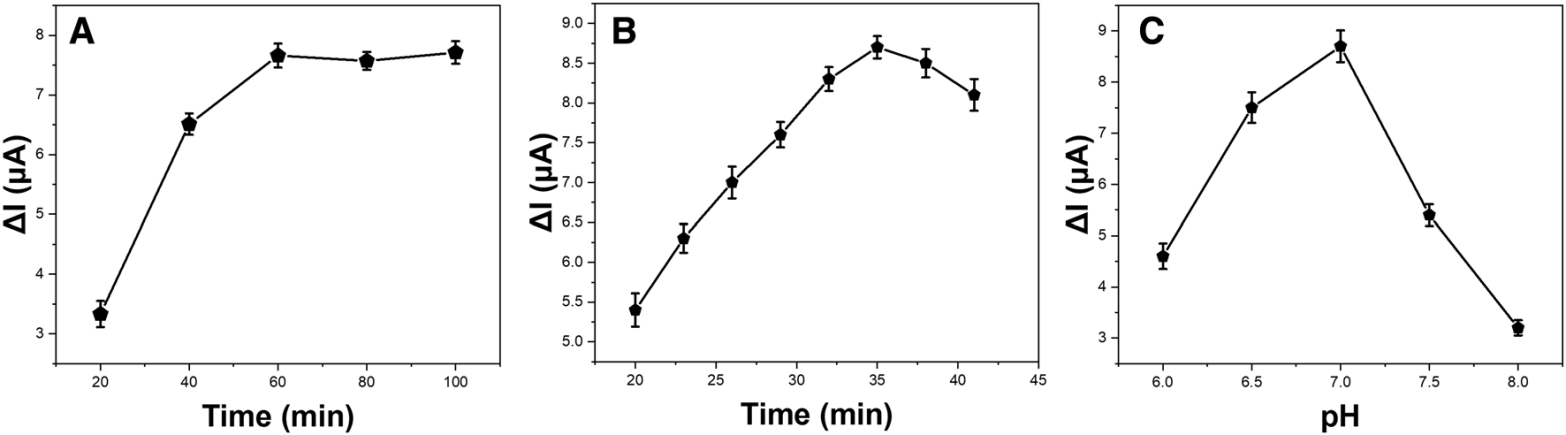
Effect of (**A**) incubation time; (**B**) incubation temperature; and (**C**) pH on the response of the immunosensor.

Key parameters including incubation time, temperature and pH were optimized to achieve maximum analytical performance of the immunosensor. Figure 7A shows the dynamic response of the immunosensor to 5 ng/mL NSE under different incubation times ranging from 20 to 100 min. The peak current change (ΔI) increased gradually with longer incubation time and reached saturation after 60 min, indicating complete immunoreaction^40^. Thus, an incubation time of 60 min was used for all measurements. The most immunocomplexes form within 15–30 min^41,42^. In our experiments, we initially tested shorter incubation times of 20, 30 and 45 min. However, the current response did not reach saturation by 30 min. This indicates that the binding interaction between anti-NSE antibodies and NSE antigens was relatively slower on the electrode interface compared to assays performed in solution phase. We hypothesize that the porous three-dimensional structure of the graphene-g-C_3_N_4_ nanocomposite results in increased surface area and binding sites for antibody immobilization. While this amplifies the overall sensor response, it likely also restricts diffusion and mass transport of antigens to some antibody binding sites.

As shown in Fig. 7B, the immunosensor response increased significantly when the incubation temperature was raised from 20 to 38 °C. This can be attributed to the fact that higher temperature facilitated faster binding kinetics between the anti-NSE antibodies and NSE antigens owing to increased molecular motion and diffusivity. However, only a slight enhancement in ΔI was observed above 38 °C. Considering potential issues with protein denaturation at excessively high temperatures which can destroy antibody binding sites and activity, 38 °C was chosen as the optimal incubation temperature.

Figure 7C depicts the effect of pH on the immunosensor response over a range between pH 6.0–8.0. The maximum ΔI value was attained around the physiological pH of 7.0. This indicates that the anti-NSE antibodies immobilized on the sensor surface retained their highest affinity and bioactivity around neutral pH, leading to most effective capture of NSE antigens from the test solutions. At lower pH conditions, the antibodies likely underwent slight conformational changes due to protonation which compromised their binding capability and capacity. Similarly, at higher pH levels the antibodies became more negatively charged, thereby electrostatically hindering their interaction with antigens. Taking into account preservation of biofunctionality, pH 7.0 offered the optimum environment for antigen–antibody binding and immunocomplex formation, generating the best immunosensor response.

### Performance of the biosensor

**Figure 8:**
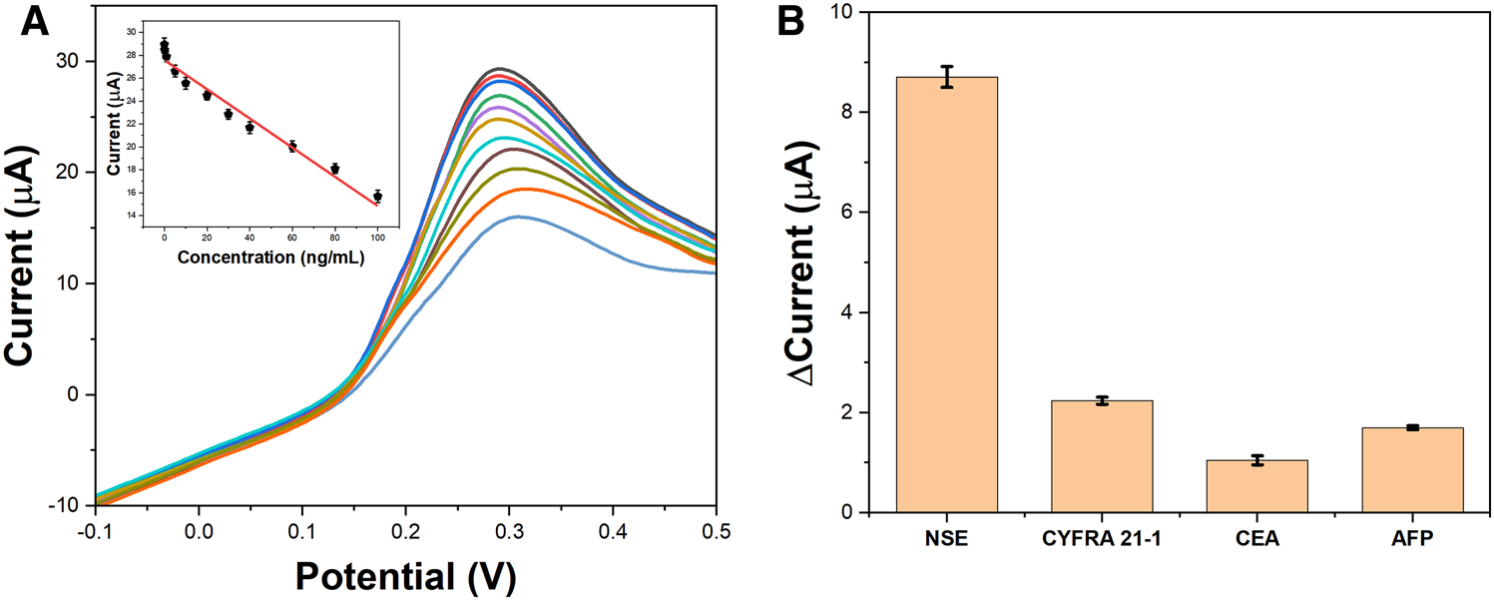
(**A**) Calibration curve of the immunosensor for different concentrations of NSE; (**B**) Selectivity of the immunosensor for 5 ng/mL NSE against other proteins.

Under optimal conditions, the immunosensor exhibited a wide linear response range from 0.01 to 100 ng/mL NSE as depicted in Fig. 8A. The regression equation was ΔI (μA) = − 0.12442 logC + 27.580 (R2 = 0.998). The limit of detection (LOD) was calculated to be 3 pg/mL NSE based on a signal-to-noise ratio of 3. Table 1 compares the analytical performance of this immunosensor with previous reports for NSE detection. The proposed platform demonstrated a much lower LOD and wider dynamic range than most other electrochemical immunosensors.

**Table 1:** Analytical properties of different electrochemical immunosensors towards NSE.

Sensor	Electrochemical technique	LDR	LOD	References
Pt skin AgPt alloy/NGR	Amperometric i-t	50 fg/mL to 100 ng/mL	18.5 fg/mL	^43^
Au–MoS_2_/MOF	Amperometric i-t	1.00 pg/mL to 100 ng/mL	0.37 pg/mL	^44^
Au/CuxO@CeO_2_	Amperometric i-t	50 fg/mL to 100 ng/mL	31.3 fg/mL	^45^
rGO/Thi/AuPt NAs	DPV	0.0001–50.0 ng/mL	0.03 pg/mL	^46^
CEA/anti-CEA/CNTs-AuNPs/GCE	CV	0.10–200 ng/mL	40 pg/mL	^47^
PPD-GR nanocomposite	PEC	1–1000 ng/mL	0.12 ng/mL	^48^
anti-NSE/g-C_3_N_4_-graphene/SPCE	DPV	0.01–100 ng/mL	3 pg/mL	This work

Selectivity experiments were performed by detecting 5 ng/mL NSE in the presence of potentially interfering proteins including cytokeratin 19 fragment (CYFRA 21-1), carcinoembryonic antigen (CEA) and alpha fetoprotein (AFP) at higher concentrations of 50 ng/mL. As shown in Fig. 8B, negligible changes in the response were observed indicating the immunosensor had excellent selectivity for NSE even in complex media. Furthermore, the reproducibility of the platform was evaluated by comparing the responses of five independently fabricated immunoelectrodes toward 5 ng/mL NSE. A relative standard deviation (RSD) of 3.2% was obtained confirming the reliability of the fabrication process and measurement protocol. The g-C_3_N_4_-graphene nanocomposite based electrochemical immunosensing platform exhibited favorable analytical properties including wide dynamic range, low detection limit, high sensitivity and selectivity for quantitative detection of the important lung cancer biomarker NSE. The simple preparation method and use of commercially available SPCEs makes this an ideal platform for point-of-care testing applications.

The applicability of the developed immunosensor for real serum matrix analysis was evaluated by performing spike and recovery experiments in human serum samples. The serum samples were diluted 100 times with 0.1 M PBS (pH 7.0) before analysis. Different concentrations of standard NSE antigen were spiked into the diluted serum samples which were then analyzed using the immunosensor.

**Figure 9:**
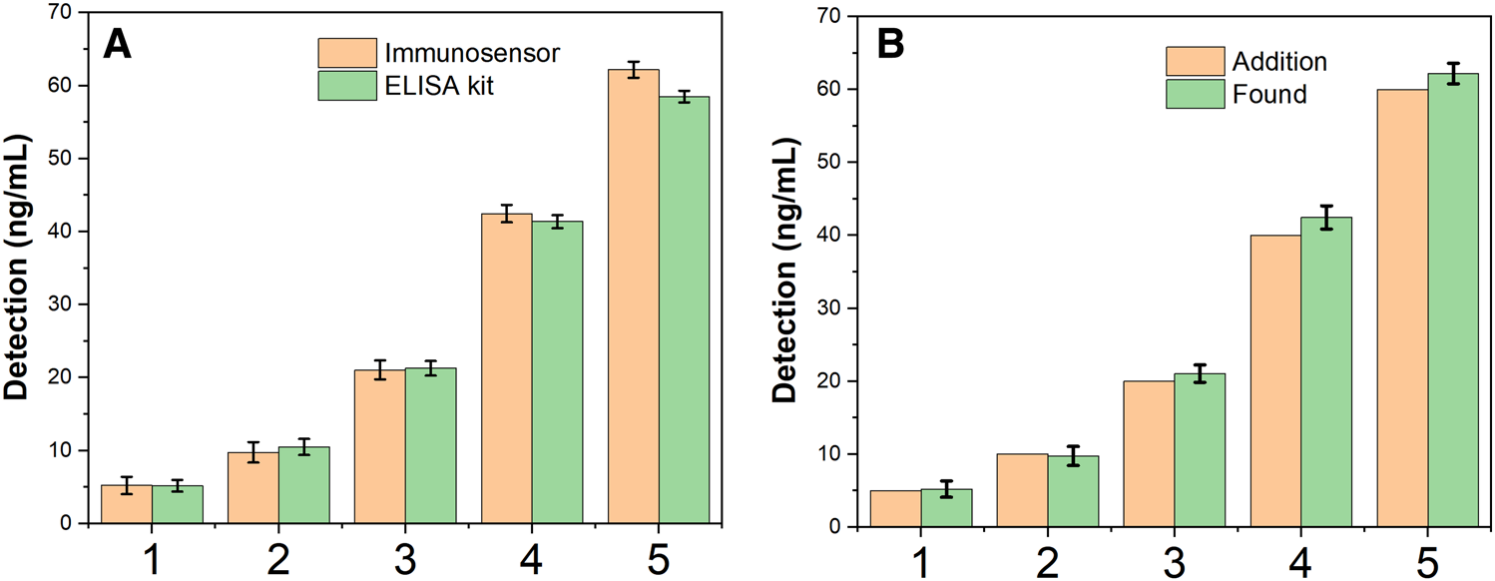
(**A**) Correlation of determined and spiked NSE concentrations in serum samples; (**B**) Comparison of NSE levels in serum samples as measured by the immunosensor and ELISA.

Table 2 summarizes the recovery results obtained at three spiking levels of NSE. The recoveries ranged from 97.60 to 106.18% with relative standard deviations below 7.66%, reflecting the accuracy of the immunosensor and the lack of matrix effects. Moreover, the determined concentrations showed good correlation with the expected values as depicted in Fig. 9A. These results indicate the immunosensor holds great promise for reliable quantification of NSE in complex clinical samples.

**Table 2:** Detection of the NSE in human serum samples with the immunosensor.

Sample	Addition (ng/mL)	Found (ng/mL)	Recovery (%)	ELISA kit	RSD (%)
1	5.00	5.22	104.4	5.17	3.33
2	10.00	9.76	97.60	10.51	2.57
3	20.00	21.05	105.25	21.28	5.05
4	40.00	42.47	106.18	41.37	7.66
5	60.00	62.20	103.67	58.51	2.54

As further validation, the serum NSE levels were measured using both the proposed immunosensor and a commercially available ELISA kit. Figure 9B shows the correlation plot between the two methods. The values exhibited good agreement with a correlation coefficient of 0.982. The relative errors were less than 8%, further confirming the potential of the immunosensor for clinical diagnosis.

The enhanced performance of the g-C_3_N_4_-graphene na nocomposite immunosensor for NSE detection can be attributed to the unique properties and synergistic effects of the composite system. First, the 2D nanosheet structure of g-C_3_N_4_ provided increased surface area for immobilization of anti-NSE antibodies leading to enhanced binding capacity. Second, g-C_3_N_4_ facilitated direct electron transfer to the redox probe due to the presence of abundant surface defects and nitrogen-induced charge delocalization. Third, the excellent conductivity of graphene nanosheets allowed rapid electron communication producing high sensitivity. Fourth, the 3D porous network formed by the two components prevented restacking and maintained active sites for unhindered biomolecule binding. Finally, the composite interface provided a biocompatible microenvironment enabling optimum antibody bioactivity.

## Conclusions

In summary, we have successfully developed a novel electrochemical immunosensing platform using a graphene-g-C_3_N_4_ nanocomposite for ultrasensitive detection of the lung cancer biomarker NSE. The g-C_3_N_4_ nanosheets and graphene nanosheets were synthesized via simple liquid exfoliation approaches and integrated together through self-assembly to form the nanocomposite material. Morphological and structural characterization confirmed the successful formation of ultrathin g-C_3_N_4_ sheets and their uniform hybridization with graphene. The nanocomposite was used to modify screen-printed carbon electrodes followed by covalent immobilization of anti-NSE antibodies to fabricate the immunosensors. The unique properties of the graphene-g-C_3_N_4_ composite facilitated efficient loading of capture antibodies while also providing a biocompatible microenvironment to retain bioactivity. In addition, the high conductivity, enlarged surface area and synergistic effects of the nanocomposite enhanced the electron transfer efficiency and electrochemical response. Systematic optimization of experimental parameters was performed to achieve maximum analytical performance. Under optimal conditions, the immunosensor exhibited a wide linear detection range from 10 pg/mL to 100 ng/mL with a low limit of detection of 3 pg/mL for NSE. The sensor also demonstrated excellent selectivity against potential interferences. Clinical sample analysis further validated the applicability of the developed immunosensing platform for sensitive and accurate quantification of NSE at clinically relevant levels. Overall, the work successfully establishes a novel graphene-g-C_3_N_4_ nanocomposite based electrochemical sensing strategy for cancer biomarker detection. With further optimization and validation, this approach holds great promise for early diagnosis of small cell lung cancer. The unique properties and synergistic effects of the integrated nanocomposite provide a versatile materials platform that can be potentially extended to electrochemical immunoassays for other protein biomarkers.

## Data Availability

Data will be available from the corresponding author upon reasonable request.
